# Role of the terminator hairpin in the biogenesis of functional Hfq-binding sRNAs

**DOI:** 10.1261/rna.060756.117

**Published:** 2017-09

**Authors:** Teppei Morita, Ryo Nishino, Hiroji Aiba

**Affiliations:** Faculty of Pharmaceutical Sciences, Suzuka University of Medical Sciences, Suzuka, Mie, 513-8670, Japan

**Keywords:** Hfq, RNA hairpin, Rho-independent terminator, bacterial sRNA, premature termination

## Abstract

Rho-independent transcription terminators of the genes encoding bacterial Hfq-binding sRNAs possess a set of seven or more T residues at the 3′ end, as noted in previous studies. Here, we have studied the role of the terminator hairpin in the biogenesis of sRNAs focusing on SgrS and RyhB in *Escherichia coli.* We constructed variant sRNA genes in which the GC-rich inverted repeat sequences are extended to stabilize the terminator hairpins. We demonstrate that the extension of the hairpin stem leads to generation of heterogeneous transcripts in which the poly(U) tail is shortened. The transcripts with shortened poly(U) tails no longer bind to Hfq and lose the ability to repress the target mRNAs. The shortened transcripts are generated in an in vitro transcription system with purified RNA polymerase, indicating that the generation of shortened transcripts is caused by premature transcription termination. We conclude that the terminator structure of sRNA genes is optimized to generate functional sRNAs. Thus, the Rho-independent terminators of sRNA genes possess two common features: a long T residue stretch that is a prerequisite for generation of functional sRNAs and a moderate strength of hairpin structure that ensures the termination at the seventh or longer position within the consecutive T stretch. The modulation of the termination position at the Rho-independent terminators is critical for biosynthesis of functional sRNAs.

## INTRODUCTION

Hfq-binding small RNAs (sRNAs), major regulatory RNAs in bacteria, are induced under specific physiological and/or stress conditions and regulate, along with an RNA chaperone Hfq, the expression of target genes at the post-transcriptional level (Waters and Storz 2009; Gottesman and Storz 2010; Vogel and Luisi 2011; Wagner and Romby 2015). The primary role of Hfq is to accelerate base-pairing between sRNAs and target mRNAs to regulate their translation. The second role of Hfq is to recruit RNase E near target mRNAs leading to rapid degradation of sRNA–mRNA hybrids (Massé et al. 2003; Morita et al. 2005). Hfq also plays a role in stabilization of sRNAs by protecting them from the attack of ribonucleases (Massé et al. 2003).

The biosynthesis of sRNAs in cells is regulated primarily at the transcription initiation step. The promoter of the individual sRNA gene is under the control of at least one transcription factor that is modulated by the cognate stress. SgrS and RyhB of *Escherichia coli* are among well-characterized sRNAs. In response to the glucose-phosphate stress, such as accumulation of glucose-6-phosphate, a transcription factor SgrR is activated to stimulate the transcription of *sgrS* encoding SgrS (Vanderpool and Gottesman 2007). The transcribed SgrS pairs with target mRNAs, such as the *ptsG* mRNA encoding the major glucose transporter, to either down- or up-regulate their expression to attenuate the glucose-phosphate stress (Vanderpool and Gottesman 2004; Morita et al. 2005; Papenfort et al. 2013). The promoter of *ryhB* encoding RyhB is under the control of the Fur repressor. Depletion of Fe^2+^ inactivates Fur, resulting in induction of RyhB, which in turn regulates the translation of several mRNAs encoding Fe-binding proteins through base-pairing (Massé and Gottesman 2002; Massé et al. 2003).

The production of sRNAs is regulated not only at the step of transcription initiation but also at the step of transcription termination (Morita et al. 2015). The sRNA genes possess a typical Rho-independent or factor-independent or intrinsic transcription terminator encoding a GC-rich RNA hairpin followed by a run of T residues (d'Aubenton Carafa et al. 1990; Ray-Soni et al. 2016). We demonstrated previously that transcription termination at the sRNA genes is enhanced under stress conditions and thereby contributes to an efficient production of active sRNAs (Morita et al. 2015). A striking feature of the Rho-independent terminators of sRNA genes is that the length of the T residue stretch is longer than seven (Otaka et al. 2011; Ishikawa et al. 2012). This feature is a prerequisite for generation of functional sRNAs because a poly(U) tail longer than seven is essential for sRNAs to bind efficiently to Hfq (Otaka et al. 2011). To generate surely the functional sRNAs, however, transcription termination must occur at the seventh or longer position within the T residue stretch by preventing “premature” termination. This implies that the modulation of the termination position at the Rho-independent terminators of sRNA genes is quite important for biosynthesis of functional sRNAs. The stability of the terminator RNA hairpin and the length of the T residue stretch are known to be major determinants for the efficiency of transcription termination, although DNA sequences around terminators also affect the termination efficiency (Lynn et al. 1988; Cheng et al. 1991; Reynolds et al. 1992; Ray-Soni et al. 2016). When the terminator hairpin is too weak, elongating RNA polymerase mostly reads through the terminator, resulting in extended forms of sRNAs, which are nonfunctional (Morita et al. 2015). On the other hand, the termination efficiency is expected to increase when the terminator hairpin is stabilized.

It is an interesting possibility that the stability of the terminator RNA hairpin affects not only the termination efficiency but also the termination position within the polythymidine stretch. The aim of the present work is to examine this possibility by using SgrS and RyhB as model sRNAs. We constructed variant *sgrS* and *ryhB* genes in which the GC-rich inverted repeat sequences of terminators are extended to stabilize the terminator hairpins. We showed by Northern blotting and 3′ RACE experiments that the extension of the stem leads to production of heterogeneous shorter transcripts possessing shortened poly(U) tails. The shortened transcripts can be generated in an in vitro system with purified RNA polymerase, confirming our working hypothesis that the stabilization of the terminator hairpin causes premature termination. We conclude that the control of the termination position within the T residue stretch is critical for the biogenesis of functional sRNAs, and that the terminator hairpin structure of sRNA genes is optimized to produce efficiently functional sRNAs under stress conditions.

## RESULTS

### Stabilization of the terminator hairpin enhances termination efficiency

**FIGURE 1.**
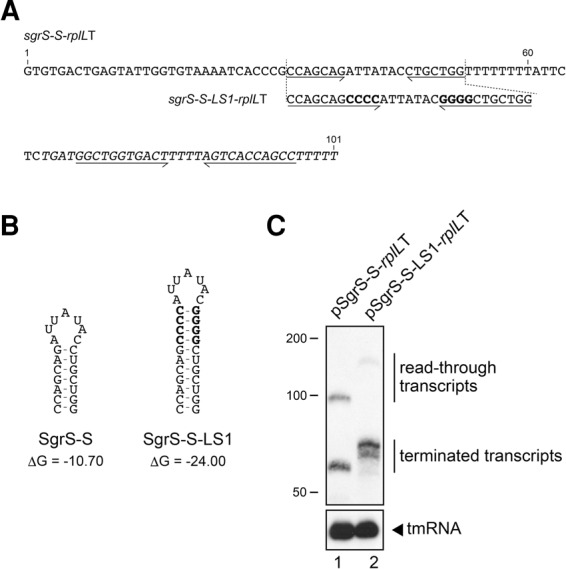
Effect of extension of the terminator stem of *sgrS* on transcription termination. (*A*) DNA sequences of *sgrS-S-rplL*T and *sgrS-S-LS1-rplL*T. The sequence corresponding to the *sgrS-S* is shown as regular letters, whereas the terminator sequence derived from the *rplL* is shown as italic letters. The inverted repeat sequences are indicated by horizontal arrows. Nucleotides are numbered from the site corresponding to the 5′ end of *sgrS-S*. The inserted sequences to stabilize the terminator stem are shown as bold letters. (*B*) The predicted secondary structures and the thermodynamic stabilities (Δ*G*, kcal/mol) of terminator RNA hairpins without poly(U) sequence were determined according to the Mfold program (Zuker 2003). (*C*) Analysis of transcription termination. TM772 (Δ*sgrS* Δ*hfq*) cells harboring indicated plasmids were grown in LB medium. At *A*_600_ = 0.6, 0.02% arabinose was added and incubation was continued for 5 min. Total RNAs were prepared, and 10 or 0.25 µg of RNA samples was subjected to Northern blotting using the SgrS-S probe and tmRNA probe, respectively.

We demonstrated previously by using a “double terminator system” that RNA polymerase frequently reads through the Rho-independent terminator of *sgrS* under normal growth conditions (Morita et al. 2015). The moderate termination efficiency of the *sgrS* terminator could be primarily due to a moderate thermodynamic stability (Δ*G*) of the terminator hairpin. If so, it is expected that stabilization of the hairpin structure of the *sgrS* terminator enhances the termination efficiency. To test this possibility, we constructed plasmid pSgrS-S-LS1-*rplL*T (“LS” stands for “long stem”) carrying the *sgrS-S*-*LS1*-*rplL*T by inserting four GC base pairs into the *sgrS* hairpin stem on plasmid pSgrS-S-*rplL*T. The full DNA sequence of *sgrS-S*-*rplL*T and the sequence around the terminator region of *sgrS-S-LS1*-*rplL*T are shown in Figure 1A. The predicted terminator RNA hairpin structures, along with their Δ*G* (kcal/mol), are shown in Figure 1B. The *sgrS-S-rplL*T is a hybrid gene in which the second terminator derived from *rplL* is placed just downstream from the *sgrS-S* (Morita et al. 2015). SgrS-S, the 3′ portion of SgrS, represents a minimal functional region of SgrS consisting of the base-pairing region and the Hfq-binding module including the Rho-independent terminator sequence (Otaka et al. 2011; Ishikawa et al. 2012). The insertion of GC pairs increases Δ*G* of the terminator RNA hairpin of SgrS-S from −10.7 to −24.0. Each plasmid was introduced into TM772 (Δ*sgrS* Δ*hfq*) cells (Table 1). The *sgrS-S* gene is under the control of an arabinose-inducible *P*_*BAD*_ promoter in these plasmids. Cells were grown in LB medium and the expression of sRNAs was induced by arabinose. Total RNAs were prepared and analyzed by Northern blotting. We first confirmed the previous observation (Morita et al. 2015) that the readthrough occurs frequently resulting in a significant amount of the readthrough product, SgrS-S-*rplL*T, along with the terminated product, SgrS-S (Fig. 1C, lane 1). When the RNAs generated from the *sgrS-S*-*LS1*-*rplL*T were analyzed, the readthrough product markedly decreased, whereas the relative abundance of terminated products increased (Fig. 1C, lane 2). This implies that stabilization of the terminator hairpin enhances the termination efficiency, as expected. Interestingly, the *sgrS-S*-*LS1*-*rplL*T generates heterogeneous shorter transcripts (Fig. 1C, lane 2), suggesting that the extension of the terminator stem causes premature termination within or before the polythymidine stretch, resulting in transcripts in which the poly(U) tail is shortened.

**TABLE 1.**
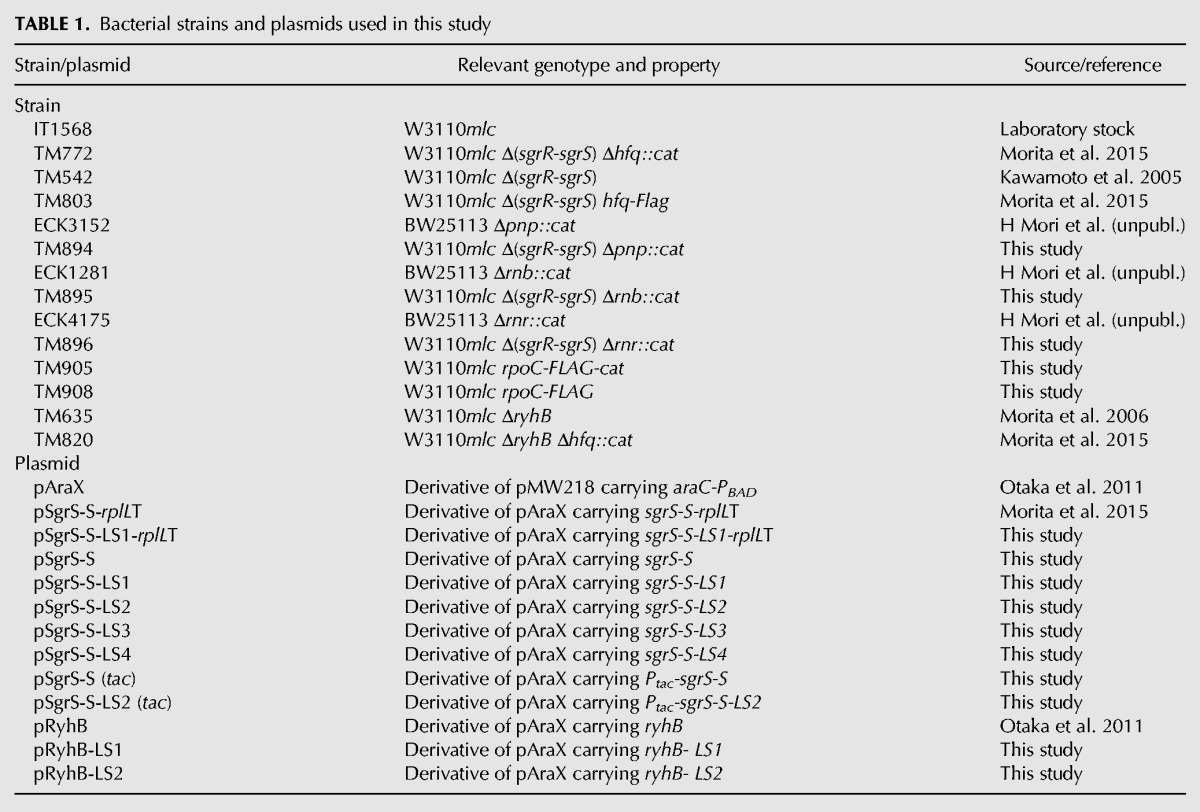
Bacterial strains and plasmids used in this study

### Generation of shorter transcripts is caused by stabilization of the terminator hairpin and enhanced by glucose-phosphate stress

**FIGURE 2.**
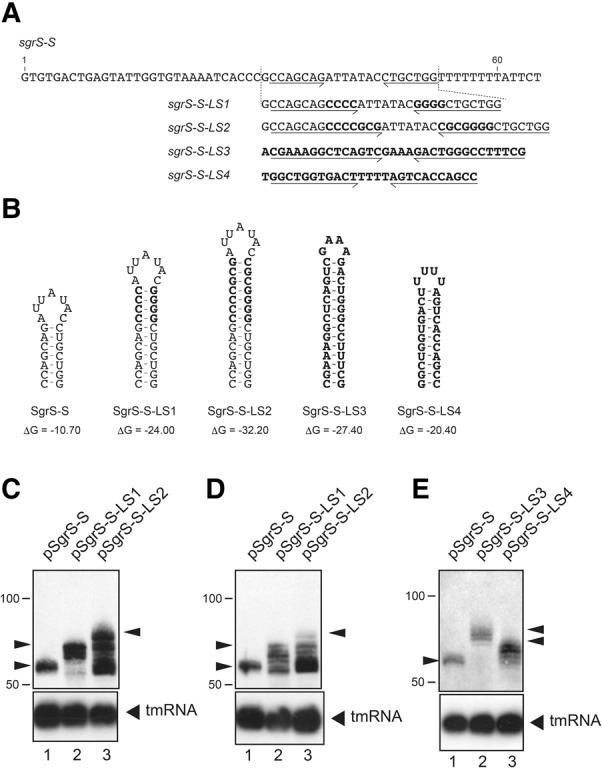
The effect of extension of the terminator stem of *sgrS-S* on the generation of heterogeneous shorter transcripts. (*A*) DNA sequences of *sgrS-S* and its variants. The inverted repeat sequences of the terminator are indicated by horizontal arrows. The inserted sequences to stabilize the terminator stem are shown as bold letters in *sgrS-S-LS1* and *sgrS-S-LS2*. The sequences derived from the *rrnB*T and *rplL*T are shown as bold letters in *sgrS-S-LS3* and *sgrS-S-LS4*, respectively. Nucleotides are numbered from the site corresponding to the 5′ end of *sgrS-S*. (*B*) The predicted secondary structures and the thermodynamic stabilities (Δ*G*, kcal/mol) of terminator RNA hairpins without a poly(U) sequence were determined according to the Mfold program (Zuker 2003). (*C*) The effect of extension of the terminator stem of *sgrS-S* on the generation of shorter transcripts. TM772 (Δ*sgrS* Δ*hfq*) cells harboring indicated plasmids were grown in LB medium. At *A*_600_ = 0.6, 0.2% arabinose was added and incubation was continued for 10 min. Total RNAs were prepared, and 20 or 0.25 µg of RNA samples was subjected to Northern blotting using the SgrS-S probe and tmRNA probe, respectively. Arrowheads represent the full-length transcripts. (*D*) The effect of extension of the terminator stem of *sgrS-S* on the generation of shorter transcripts under glucose-phosphate stress. TM772 (Δ*sgrS* Δ*hfq*) cells harboring indicated plasmids were grown in LB medium. At *A*_600_ = 0.6, 0.01% αMG was added and incubation was continued for 10 min. Then, 0.2% arabinose was added and incubation was continued for 10 min. Total RNAs were prepared, and 20 or 0.25 µg of RNA samples was subjected to Northern blotting using the SgrS-S probe and tmRNA probe, respectively. Arrowheads represent the full-length transcripts. (*E*) The effect of replacement of the terminator hairpin of *sgrS-S* with those derived from *rrnB*T and *rplL*T on the generation of shorter transcripts. TM772 (Δ*sgrS* Δ*hfq*) cells harboring indicated plasmids were grown in LB medium and treated as described in *D*. Total RNAs were prepared, and 20 or 0.25 µg of RNA samples was subjected to Northern blotting using the SgrS-S probe and tmRNA probe, respectively. Arrowheads represent the full-length transcripts.

To focus more on the effect of the strength of the terminator hairpin on the generation of the heterogeneous shorter transcripts, we constructed plasmid pSgrS-S-LS1 carrying the *sgrS-S*-*LS1* by inserting four GC base pairs into the hairpin stem of the *sgrS-S* on plasmid pSgrS-S (Fig. 2A). We also constructed plasmid pSgrS-S-LS2 carrying the *sgrS-S*-*LS2* by inserting seven GC base pairs (Fig. 2A). The Δ*G* of the RNA hairpin of SgrS-S-LS1 is –24.0, whereas that of SgrS-S-LS2 is –32.2 (Fig. 2B). Each plasmid was introduced into TM772 (Δ*sgrS* Δ*hfq*) cells. Cells were grown in LB medium and expression of sRNAs was induced by arabinose. Total RNAs were prepared and analyzed by Northern blotting. The *sgrS-S*-*LS1* generated heterogeneous shorter transcripts along with the full-length SgrS-S-LS1 as expected (Fig. 2C, lane 2). The generation of heterogeneous shorter transcripts from the *sgrS-S*-*LS2* (lane 3) was more significant compared with the *sgrS-S*-*LS1*, indicating that the extent of the production of the heterogeneous shorter transcripts correlates with the strength of the terminator hairpin.

Because transcription termination is enhanced under stress conditions (Morita et al. 2015), we assume that the glucose-phosphate stress may also enhance the production of shorter transcripts. Then, we tested the effect of glucose-phosphate stress on the generation of shorter transcripts at the *sgrS* terminator by using Δ*sgrS* Δ*hfq* cells harboring either pSgrS-S, pSgrS-S-LS1, or pSgrS-S-LS2. Cells were grown in LB medium to exponential phase and exposed to nonmetabolizable glucose analog α-methylglucoside (αMG), and then to arabinose. Total RNAs were prepared and subjected to Northern blotting using the SgrS-S probe (Fig. 2D). Both the *sgrS-S*-*LS1* and *sgrS-S*-*LS2* generated the heterogeneous shorter transcripts along with the full-length transcripts (Fig. 2D, lanes 2,3). Again, the generation of shorter transcripts from the *sgrS-S*-*LS2* was more significant compared with the *sgrS-S*-*LS1*. In addition, the production of shorter transcripts from the *sgrS-S*-*LS1* and *sgrS-S*-*LS2* was clearly enhanced under the glucose-phosphate stress. The generation of shorter transcripts was not observed from the wild-type *sgrS-S* even under the stress condition (Fig. 2D, lane 1).

We also tested the effect of replacing the terminator hairpin of *sgrS-S* with the corresponding portion derived from strong Rho-independent terminators. For this, we constructed plasmid pSgrS-S-LS3 and pSgrS-S-LS4 in which the terminator hairpin was replaced with those derived from *rrnB* and *rplL*, respectively (Fig. 2A). The thermodynamic stabilities of these hairpins are −27.4 and −20.4, respectively (Fig. 2B). Cells harboring each plasmid were grown in LB medium to exponential phase and exposed to αMG, and then to arabinose. Total RNAs were prepared and subjected to Northern blotting using the SgrS-S probe (Fig. 2E). The data indicate that heterogeneous shorter transcripts are generated efficiently from pSgrS-S-LS3 and pSgrS-S-LS4 (Fig. 2E, lanes 2,3). This suggests that the stabilization of the terminator hairpin itself rather than specific sequences within the hairpin is responsible for the generation of heterogeneous shorter transcripts.

### Properties of the heterogeneous shorter transcripts

**FIGURE 3.**
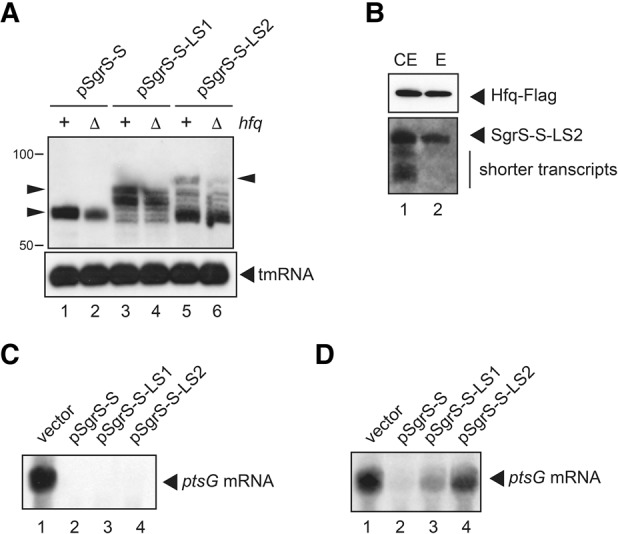
Properties of heterogeneous transcripts. (*A*) The effect of *hfq* backgrounds on the expression of heterogeneous transcripts. TM542 (Δ*sgrS*) and TM772 (Δ*sgrS* Δ*hfq*) cells harboring indicated plasmids were grown in LB medium. At *A*_600_ = 0.6, 0.1% αMG was added and incubation was continued for 10 min, and then 0.02% arabinose was added and incubation was continued for 5 min. Total RNAs were prepared, and 10 or 0.25 µg of RNA samples was subjected to Northern blotting using the SgrS-S probe and tmRNA probe, respectively. Arrowheads represent the full-length transcripts. (*B*) An examination of Hfq binding of heterogeneous transcripts. TM803 (Δ*sgrS hfq-Flag*) cells harboring pSgrS-S-LS2 were grown in LB medium. At *A*_600_ = 0.6, 0.1% αMG was added and incubation was continued for 10 min, and then 0.02% arabinose was added and incubation was continued for 5 min. Crude extract was prepared and subjected to the pull-down assay using anti-Flag agarose as described in Materials and Methods. (*C*,*D*) The effect of extension of the terminator stem of *sgrS-S* on SgrS function in the presence (*D*) and absence (*C*) of glucose-phosphate stress. TM542 (Δ*sgrS*) cells harboring indicated plasmids were grown in LB medium. To prepare total RNAs from cells without the stress, 0.02% arabinose was added at *A*_600_ = 0.6 and incubation was continued for 5 min (*C*). To prepare total RNAs from cells with the stress, the culture of *A*_600_ = 0.6 was exposed to 0.1% αMG for 10 min, and then 0.02% arabinose for 5 min (*D*). Ten micrograms of RNA samples was subjected to Northern blotting using the *ptsG* probe.

We expect that the poly(U) tail is shortened in the heterogeneous shorter transcripts. If so, the levels of shorter transcripts would not be affected by the *hfq* backgrounds because SgrS-S possessing a shorter poly(U) tail no longer binds to Hfq (Otaka et al. 2011). Then, we investigated the expression of SgrS-S in TM542 (*hfq*^+^) and TM772 (Δ*hfq*) cells (Table 1) under the glucose-phosphate stress condition in which the generation of shorter transcripts is enhanced. The abundance of SgrS-S increased in *hfq*^+^ cells compared with in Δ*hfq* cells (Fig. 3A, lanes 1,2). On the other hand, the levels of shorter transcripts were not affected by the *hfq* backgrounds, whereas the abundance of full-length SgrS-S-LS1 and SgrS-S-LS2 was significantly elevated in *hfq*^+^ cells (Fig. 3A, lanes 3–6). This strongly suggests that the shorter transcripts lose the ability to bind to Hfq.

To examine directly the Hfq-binding ability of shorter transcripts, we carried out a pull-down assay using cell extracts of *hfq-Flag* cells harboring pSgrS-S-LS2. Cell extracts were incubated with anti-Flag M2-agarose beads. Proteins bound to the agarose beads were analyzed by Western blotting using anti-Flag antibodies. The affinity-purified Hfq-Flag was treated with phenol and subjected to Northern blotting. As shown in Figure 3B, the full-length SgrS-S-LS2 but not shorter transcripts copurified with Hfq-Flag. This indicates that the shorter transcripts generated from the *sgrS-S-LS2* are not able to bind to Hfq. Thus, it is highly likely that the poly(U) tail is shortened in the heterogeneous shorter transcripts.

The abundance of full-length active SgrS-S variants is markedly decreased with accumulation of inactive shorter transcripts in cells harboring pSgrS-S-LS1 or pSgrS-S-LS2, in particular under the glucose-phosphate stress. Thus, it is expected that the down-regulation of *ptsG* mRNA, the target of SgrS, is impaired in these cells. To examine this, we performed Northern blot analysis by using the *ptsG* probe. First, we analyzed the expression of *ptsG* mRNA in cells harboring the vector plasmid pAraX, pSgrS-S, pSgrS-S-LS1, or pSgrS-S-LS2 under normal growth conditions (Fig. 3C). The *ptsG* mRNA was highly expressed in cells harboring pAraX (Fig. 3C, lane 1), whereas the abundance of the full-length *ptsG* mRNA was dramatically reduced when SgrS-S was expressed (Fig. 3C, lane 2) because SgrS-S accelerates the RNase E-dependent degradation of *ptsG* mRNA (Morita et al. 2005). The down-regulation of *ptsG* mRNA was also observed when SgrS-S-LS1 or SgrS-S-LS2 was expressed (Fig. 3C, lanes 3,4). These results suggest that the abundance of the full-length SgrS-S-LS1 and SgrS-S-LS2 is still sufficient to down-regulate the *ptsG* mRNA under normal growth conditions. Then, we carried out Northern analysis by using RNA samples prepared from cells exposed to the glucose-phosphate stress (Fig. 3D). The expression of *ptsG* mRNA was not affected by the stress in cells harboring pAraX (Fig. 3D, lane 1). The dramatic reduction of the *ptsG* mRNA was observed in cells harboring pSgrS-S as expected (Fig. 3D, lane 2). On the other hand, the abundance of *ptsG* mRNA clearly increased in cells harboring pSgrS-S-LS1 or pSgrS-S-LS2 under glucose-phosphate stress (Fig. 3D, lanes 3,4), indicating that the down-regulation of *ptsG* mRNA was partially suppressed in these cells. It is apparent that the marked reduction of the active full-length SgrS-S variants is responsible for this suppression of the down-regulation of *ptsG* mRNA.

### Analysis of 3′ ends of transcripts

**FIGURE 4.**
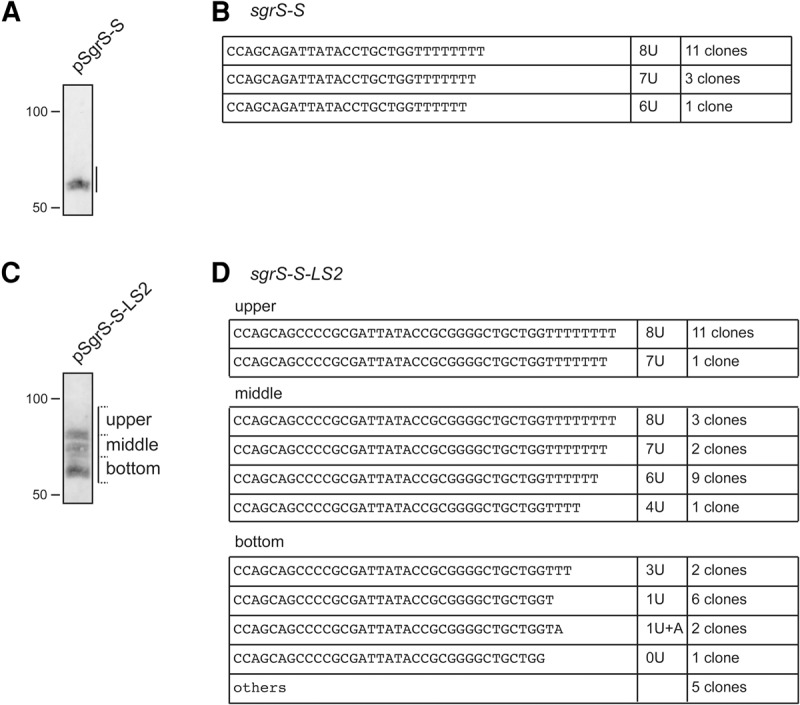
Analysis of the 3′ ends of transcripts derived from *sgrS-S* and *sgrS-S-LS2*. TM542 (Δ*sgrS*) cells harboring pSgrS-S (*A*) or pSgrS-S-LS2 (*C*) were grown in LB medium. At *A*_600_ = 0.6, 0.1% αMG was added and incubation was continued for 10 min, and then 0.02% arabinose was added and incubation was continued for 5 min. Total RNAs were prepared and duplicate RNA samples (10 µg) were resolved side-by-side on a 12% polyacrylamide gel electrophoresis in the presence of 8 M urea. One side of the gel was subjected to Northern blotting (*A*,*C*). The region corresponding to transcripts was cut out from the other side of the gel (*A*,*C*). A single gel piece containing the SgrS-S band was used for transcripts from *sgrS-S (A)*. The gel was divided into three gel pieces (*upper*, *middle*, and *lower*) for transcripts from *sgrS-S-LS2* (*C*). The *upper* gel piece corresponds to the full-length transcript, whereas the *middle* and *lower* gel pieces correspond to heterogeneous shorter transcripts. RNAs were purified from the gel pieces and subjected to 3′-RACE analysis. DNA sequences corresponding to the 3′ region of transcripts were determined by using randomly picked-up plasmid clones containing amplified cDNAs. The sequences and number of clones analyzed are shown (*B*,*D*). Others represent the clones in which the 3′ ends of RNAs were mapped in the region prior to the terminator T residue stretch (*D*).

To verify that the poly(U) tail is indeed shortened in the heterogeneous shorter transcripts, we determined the 3′ ends of RNAs generated from the *sgrS-S*-*LS2* in *hfq*^+^ cells under the glucose-phosphate stress by the 3′-RACE (rapid amplification of cDNA ends) experiment (Argaman et al. 2001; Kawano et al. 2005). In this experiment, total cellular RNAs and a linker DNA oligo are ligated in vitro and the RNA–DNA junction region is amplified by RT-PCR. The amplified cDNA fragments were cloned into a plasmid and subjected to DNA sequence analysis. The 3′ ends of transcripts can be determined by finding the junction between RNAs and the 5′ end of ligated linker oligo DNA. We first analyzed the 3′ ends of transcripts generated from the *sgrS-S*. The total RNAs were separated by electrophoresis on a polyacrylamide gel containing 8 M urea. The gel region corresponding to SgrS-S was cut out and RNAs were purified from the gel piece (Fig. 4A). The purified RNAs were subjected to the 3′-RACE experiment. The sequences around the junction region of randomly isolated 15 cDNA clones indicate that 11, three, and one clones correspond to SgrS-S possessing 8U, 7U, and 6U tail, respectively (Fig. 4B). Thus, the *sgrS-S* is able to produce quite efficiently active SgrS-S possessing a long poly(U) tail. Then, we analyzed the 3′ ends of transcripts generated from the *sgrS-S*-*LS2.* The gel region corresponding to heterogeneous RNA bands was divided into three pieces as shown in Figure 4C. RNAs prepared from each piece were subjected to the 3′-RACE experiment (Fig. 4D). The sequence analysis of randomly isolated cDNA clones indicates that RNAs derived from the upper region exclusively correspond to SgrS-S-LS2 possessing a long poly(U) tail consisting of eight or seven uridine residues. The length of the poly(U) tail of RNAs derived from the middle region varies from four to eight. The length of the poly(U) tail of RNAs derived from the lower region was <3. The 3′ ends of several RNAs obtained from the lower region were mapped in the *sgrS* terminator region preceding the poly(U) tail. Thus, we conclude that the poly(U) tail of the heterogeneous shorter transcripts is indeed shortened.

### Effect of 3′ exoribonuclease on the generation of shorter transcripts and the stability of transcripts

**FIGURE 5.**
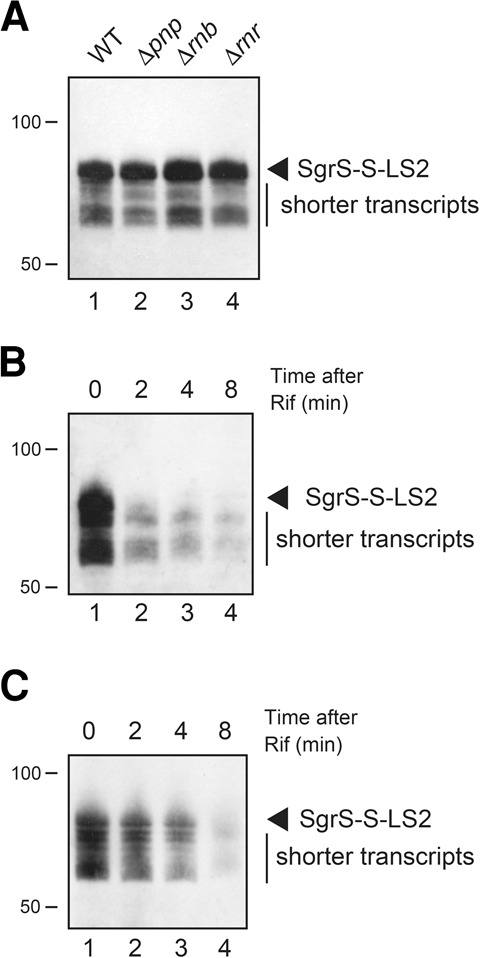
(*A*) Effect of mutation in 3′ exoribonuclease genes on generation of shorter transcripts. TM542 (Δ*sgrS*), TM894 (Δ*sgrS* Δ*pnp*), TM895 (Δ*sgrS* Δ*rnb*), and TM896 (Δ*sgrS* Δ*rnr*) cells harboring pSgrS-S-LS2 were grown in LB medium containing 0.2% arabinose. At *A*_600_ = 0.6, total RNAs were prepared and 10 µg of RNA samples was subjected to Northern blotting using the SgrS-S probe. (*B*,*C*) Stability of heterogeneous transcripts in the presence (*C*) and absence (*B*) of stress. TM772 (Δ*sgrS* Δ*hfq*) cells harboring pSgrS-S-LS2 were grown in LB medium. For the preparation of samples in the absence of stress, 0.2% arabinose was added at *A*_600_ = 0.6 and incubation was continued for 10 min, and then rifampicin (250 µg/mL) was added. Total RNAs were prepared at the indicated time after the addition of rifampicin. For the preparation of samples in the presence of stress, cells were exposed to 0.01% αMG for 10 min prior to the addition of arabinose. The RNA samples (20 µg) were subjected to Northern blotting using the SgrS-S. Arrowheads represent the full-length transcripts.

Our hypothesis is that the shorter transcripts are generated by premature transcription termination within or before the terminator polythymidine stretch. However, it is also possible that the shorter products are generated through trimming of the primary transcripts by 3′ exoribonucleases. To test this possibility, we investigated the expression of *sgrS-S*-*LS2* in cells lacking either PNPase (*pnp*), RNase II (*rnb*), or RNase R (*rnr*), respectively. The production of shorter transcripts was not significantly affected by the individual mutation (Fig. 5A). Although this result is consistent with our hypothesis, it does not prove that the shorter transcripts are generated primarily by premature termination because we do not rule out the involvement of 3′ exonucleases based on the results from *pnp, rnb,* and *rnr* single mutants.

We also performed a chase experiment to examine the fate of the heterogeneous transcripts in Δ*sgrS* Δ*hfq* cells harboring pSgrS-S-LS2. Cells were grown to exponential phase, and expression of sRNAs was induced by arabinose. Rifampicin was added to prevent further initiation of transcription. RNAs were isolated at various times after the addition of rifampicin, and subjected to Northern blotting (Fig. 5B). The stability of shorter transcripts was essentially the same as that of the full-length transcripts. As expected, the full-length transcript but not the heterogeneous transcripts were markedly stabilized in the *hfq*^+^ background (data not shown). We also examined the fate of the heterogeneous transcripts under the glucose-phosphate stress. In this case, cells were exposed to αMG prior to the addition of arabinose. Again, the stability of shorter transcripts was the same as that of the full-length transcripts, although both transcripts were significantly stabilized by the stress (Fig. 5C). These results suggest that the shorter transcripts are generated by transcription termination rather than by degradation of primary transcripts.

### The shortened transcripts are generated in vitro with purified RNA polymerase

**FIGURE 6.**
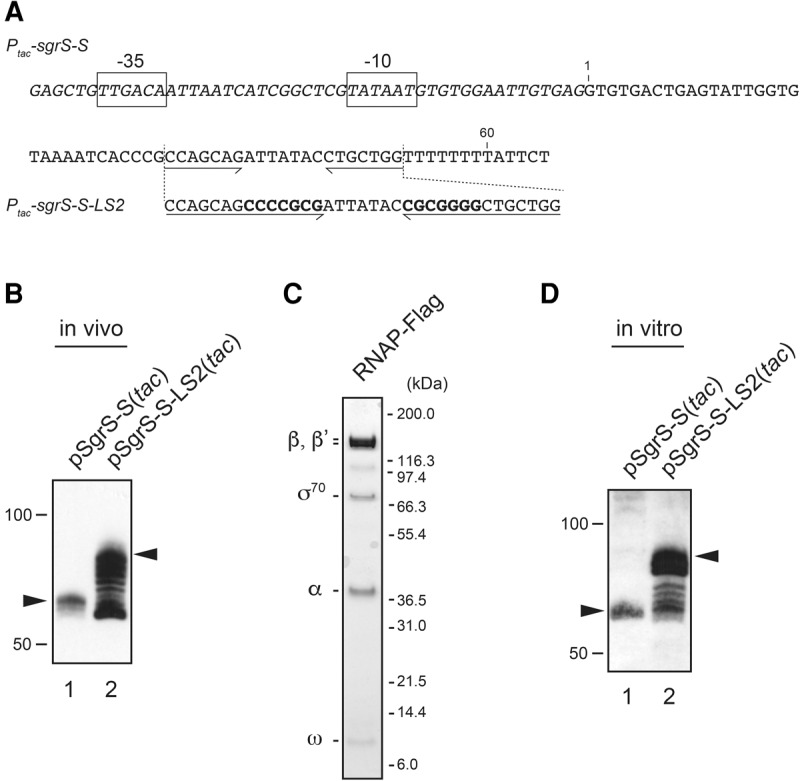
Analysis of transcription of *sgrS-S* and *sgrS-S-LS2* directed by the *tac* promoter. (*A*) DNA sequences of the chimeric *P*_*tac*_-*sgrS-S* and *P_tac_-sgrS-S-LS2* genes. The sequence corresponding to *sgrS-S* is shown as regular letters, whereas the *tac* promoter sequence is shown as italic letters. The sequences of the −10 and −35 regions of the *tac* promoter are boxed. The inverted repeat sequences of the *sgrS-S* terminator are indicated by horizontal arrows. The inserted sequences to stabilize the terminator stem are shown as bold letters. (*B*) In vivo expression of the *P*_*tac*_-*sgrS-S* and *P*_*tac*_-*sgrS-S-LS2.* TM772 (Δ*sgrS* Δ*hfq*) cells harboring indicated plasmids were grown in LB medium. Total RNAs were prepared at *A*_600_ = 0.4, and 20 µg of RNA samples was subjected to Northern blotting using the SgrS-S probe. Arrowheads represent the full-length transcripts. (*C*) SDS-PAGE analysis of the purified RNAP-Flag. The affinity-purified PNAP-Flag sample (5 µL) was analyzed by SDS-PAGE and Coomassie staining as described in Materials and Methods. The bands corresponding to RNAP subunits are indicated on the *left*. Protein size markers are shown on the *right*. (*D*) In vitro transcription of the *P*_*tac*_-*sgrS-S* and *P*_*tac*_-*sgrS-S-LS2*. The transcription reaction was performed as described in Materials and Methods. The samples were subjected to Northern blotting using the SgrS-S probe. Arrowheads represent the full-length transcripts.

To verify directly that the shortened transcripts are generated by premature termination, we tried to perform an in vitro transcription assay. For this, we constructed plasmids, pSgrS-S(*tac*) and pSgrS-S-LS2(*tac*), in which the *sgrS-S* and the *sgrS-S*-*LS2* are placed under the constitutive *tac* promoter (*P*_*tac*_), respectively (Fig. 6A). Each plasmid was introduced into Δ*sgrS* Δ*hfq* cells. Cells were grown in LB medium, and total RNAs were prepared and analyzed by Northern blotting. As expected, the full-length SgrS-S was expressed in cells carrying pSgrS-S(*tac*), whereas heterogeneous shorter transcripts along with the full-length SgrS-S-LS2 were generated in cells carrying pSgrS-S-LS2(*tac*) (Fig. 6B). We purified Flag-tagged RNA polymerase (RNAP-Flag) from the strain carrying the *rpoC-Flag* allele (Fig. 6C). Then, we carried out an in vitro transcription experiment using RNAP-Flag, pSgrS-S(*tac*), or pSgrS-S-LS2(*tac*), and four nucleoside triphosphates (NTPs). If our hypothesis is correct, pSgrS-S-LS2(*tac*) should produce prematurely terminated transcripts. After the transcription reaction, transcripts were subjected to Northern blotting using the SgrS-S probe (Fig. 6D). When pSgrS-S(*tac*) was used as a DNA template, a transcript corresponding to the full-length SgrS-S generated in vivo was detected as a major RNA band (Fig. 6D, lane 1). Importantly, the heterogeneous shorter transcripts that are similar to those generated in vivo were produced when pSgrS-S-LS2(*tac*) was used as a template (Fig. 6D, lane 2). Thus, generation of the heterogeneous transcripts with shortened poly(U) tails is ascribed at least partly to termination events occurring earlier in the run of T residues.

### Effect of stabilization of the *ryhB* terminator hairpin on expression of RyhB and *sodB* mRNA

**FIGURE 7.**
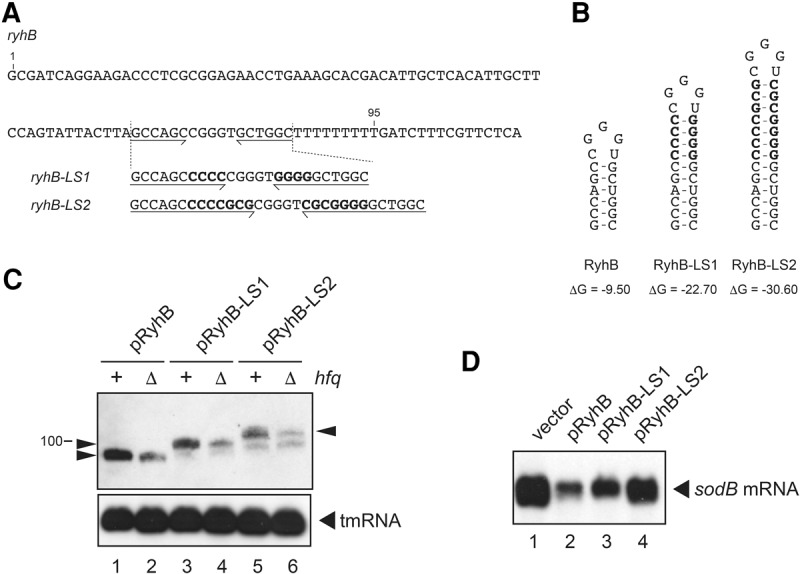
The effect of extension of the terminator stem of *ryhB* on transcription termination and on properties of RyhB. (*A*) DNA sequences of *ryhB* and its variants. The inverted repeat sequences of terminators are indicated by horizontal arrows. The inserted sequences to stabilize the terminator stem are shown as bold letters. Nucleotides are numbered from the site corresponding to the 5′ end of *ryhB*. (*B*) The predicted secondary structures and the thermodynamic stabilities (Δ*G*, kcal/mol) of RNA hairpins without the poly(U) sequence were determined according to the Mfold program (Zuker 2003). (*C*) Generation of heterogeneous transcripts and effect of *hfq* backgrounds. TM635 (Δ*ryhB*) and TM820 (Δ*ryhB* Δ*hfq*) cells harboring the indicated plasmid were grown in LB medium. At *A*_600_ = 0.6, 250 µM of 2, 2′-dipyridyl was added and incubation was continued for 10 min. Then, 0.02% arabinose was added and incubation was continued for 5 min. Total RNAs were prepared, and 1.6 or 0.25 µg of RNA samples was subjected to Northern blotting using the RyhB probe and tmRNA probe, respectively. Arrowheads represent the full-length transcripts. (*D*) Effect of extension of the terminator stem on RyhB function. Two micrograms of RNA samples described in Figure 7C was subjected to Northern blotting using the *sodB* probe.

We also examined the effect of stabilization of the terminator hairpin of the *ryhB* on expression of RyhB. To do this, we constructed plasmid pRyhB-LS1 carrying the *ryhB-LS1* by inserting four GC base pairs into the hairpin stem of the *ryhB* on plasmid pRyhB (Fig. 7A). We also constructed plasmid pRyhB-LS2 carrying the *ryhB*-*LS2* by inserting seven GC base pairs into the hairpin stem of the *ryhB* (Fig. 7A). The predicted RNA hairpin structures and the Δ*G* values are shown in Figure 7B. Each plasmid was introduced into TM635 (Δ*ryhB hfq*^+^) and TM820 (Δ*ryhB*Δ*hfq*) cells. Cells were grown in LB medium to exponential phase and exposed to 2,2′-dipyridyl to deplete Fe^2+^, and then arabinose was added to induce the transcription of *ryhB*. Expression of RyhB and its variants was analyzed by Northern blotting. As shown in Figure 7C, shorter transcripts, along with a decreasing amount of the full-length transcripts, were generated from the *ryhB-LS1* and the *ryhB-LS2* but not from the wild-type *ryhB* genes. The generation of shorter transcripts was more significant in the case of *ryhB-LS2* compared to *ryhB-LS1*. Consistent with the increase in shorter transcripts, the amount of the full-length transcripts decreases more significantly in the *ryhB-LS2*. The abundance of the full-length RyhB, RyhB-LS1, and RyhB-LS2 in the *hfq*^+^ cells was significantly higher than that in the Δ*hfq* cells, reflecting Hfq binding of the full-length RyhB variants. On the other hand, the *hfq* backgrounds did not affect the level of the shorter transcripts, suggesting that the poly(U) tails of these RyhB variants are shortened. Then, we examined the expression of *sodB* mRNA in the *hfq*^+^ cells harboring the vector plasmid pAraX, RyhB, RyhB-LS1, or RyhB-LS2 under the Fe^2+^ depletion. The *sodB* mRNA is well expressed in cells harboring pAraX (Fig. 7D, lane 1). A marked reduction of the *sodB* mRNA was observed in cells harboring pRyhB (Fig. 7D, lane 2). The reduction of *sodB* mRNA expression was moderate in cells harboring pRyhB-LS1 or pRyhB-LS2 (Fig. 7D, lanes 3,4), indicating that the down-regulation of *sodB* mRNA was partially suppressed in these cells. The suppression of the *sodB* mRNA down-regulation was stronger in cells harboring pRyhB-LS2 in which the reduction of levels of the full-length active RyhB variant was more significant. Thus, we conclude that the stabilization of the terminator hairpin of the *ryhB* also leads to generation of the shortened transcript with concomitant reduction of the active full-length transcript.

## DISCUSSION

The poly(U) sequence at the 3′ end of sRNAs is a central element of the Hfq-binding module of sRNAs (Otaka et al. 2011; Ishikawa et al. 2012). Hfq hexamer binds to the poly(U) tail through its proximal face (Sauer and Weichenrieder 2011; Schu et al. 2015). We have previously found that the poly(U) tail must be seven residues or longer to achieve a stable binding to Hfq that is required for the regulatory function of sRNAs (Otaka et al. 2011). Therefore, terminators of sRNA genes are expected to fulfill the structural features that ensure the generation of functional sRNAs possessing a long poly(U) tail. In fact, the polythymidine stretch of Rho-independent terminators of sRNA genes are longer than seven (Otaka et al. 2011; Ishikawa et al. 2012), whereas those of many other genes vary from four to eight or more (d'Aubenton Carafa et al. 1990). A recent global study on sRNA–target interactions has also revealed that the poly(U) tails of Rho-independent terminators are longer than those of mRNAs (Melamed et al. 2016). The presence of a long polythymidine stretch in the terminator is essential but not sufficient for generation of the functional sRNAs. To generate the functional sRNAs, the consecutive terminator A residues on the template strand must be transcribed up to the seventh position before termination occurs within the T residue stretch. Thus, the modulation of the termination position at the Rho-independent terminators of sRNA genes is critical for the biogenesis of sRNAs. We assumed that thermodynamic stability of the terminator RNA hairpin affects the termination position within the polythymidine stretch and therefore acts as a key element for generation of functional sRNAs.

In the present work, we studied the role of the terminator hairpin in the biogenesis of sRNAs, focusing on how the hairpin stability affects the termination position at Rho-independent terminators. We constructed variant *sgrS* and *ryhB* genes in which the inverted repeat sequences of terminators are extended to stabilize the terminator hairpin. The stabilization of a hairpin at a given terminator is expected to enhance the termination efficiency because the strength of the terminator hairpin, along with the length of the T residue stretch, is an important element to determine the efficiency of termination at Rho-independent terminators, although the DNA sequences within and near the hairpin also affect the termination efficiency (Lynn et al. 1988; Cheng et al. 1991; Reynolds et al. 1992; Ray-Soni et al. 2016). Indeed, we showed that the extension of the terminator stem markedly enhances termination at the *sgrS* terminator (Fig. 1). The central finding in the present study is that the extension of the stem leads to generation of significant amounts of heterogeneous transcripts in which the poly(U) tail is shortened (Figs. 1–4, 6, 7). The transcripts with shortened poly(U) tails lose the ability to bind to Hfq and therefore are no longer able to repress target mRNAs (Figs. 3, 7). Importantly, we found that heterogeneous transcripts can be generated in an in vitro transcription system with purified RNA polymerase (Fig. 6), confirming that premature transcription termination within and/or before the terminator polythymidine stretch is at least partly responsible for the generation of shortened transcripts. Thus, the stability of the terminator RNA hairpins of *sgrS* and *ryhB* genes is optimized (not too strong and not too weak) to ensure an effective termination at the seventh or longer position of the consecutive T stretch. The moderate thermodynamic stability of the terminator hairpin is associated with most well-characterized sRNAs (Table 2), whereas the hairpin stability fluctuates more widely in many other terminators (d'Aubenton Carafa et al. 1990). The present study, along with previous studies (Otaka et al. 2011; Ishikawa et al. 2012; Morita et al. 2015), led us to conclude that the Rho-independent terminators of sRNA genes possess two common features to generate functional sRNAs: a long T residue stretch (longer than seven) and a moderate strength of hairpin structure that allows the termination at the seventh or longer position of the consecutive T stretch.

**TABLE 2.**
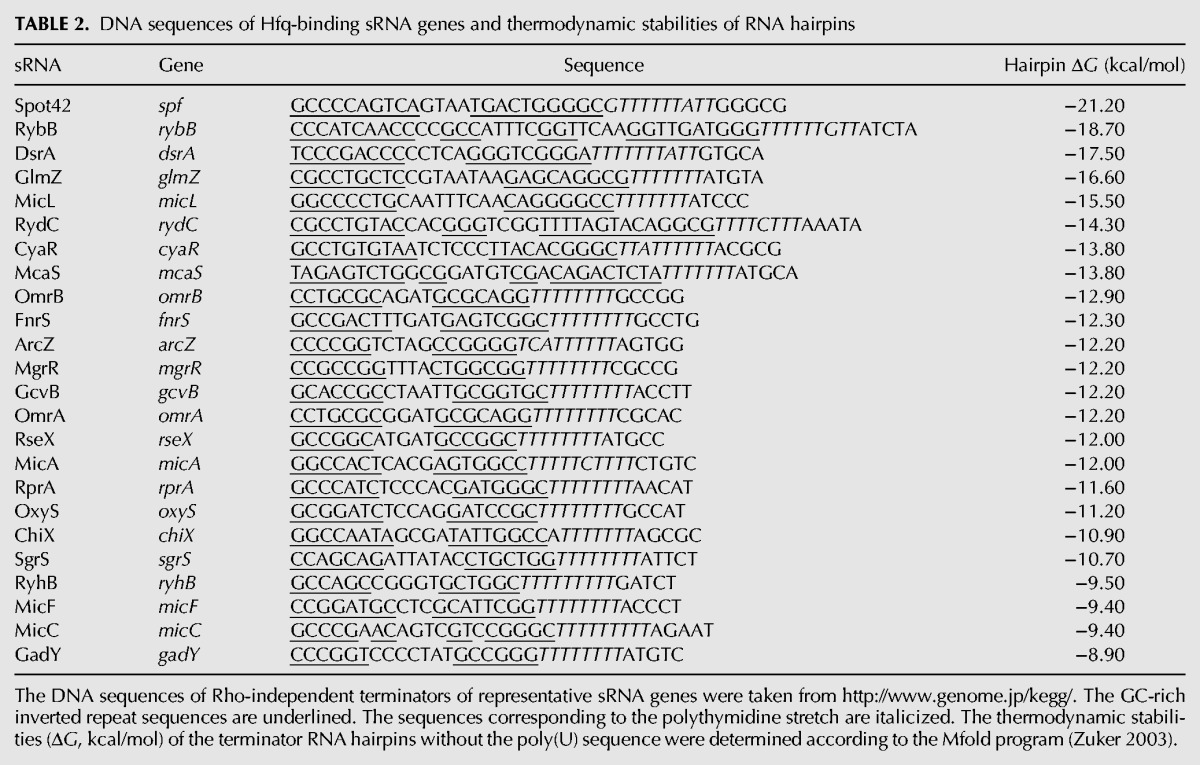
DNA sequences of Hfq-binding sRNA genes and thermodynamic stabilities of RNA hairpins

Concerning the roles of the terminator hairpin and the T residue stretch in transcription termination at Rho-independent terminators, it was originally proposed that the formation of the RNA hairpin induces pausing of the elongation complex, and the low stability of rU–dA pairs allows the release of the nascent transcript and RNA polymerase from the template DNA (Rosenberg and Court 1979; Platt 1986; Yager and von Hippel 1991). The later study suggests that the U-tract is principally responsible for the pause prior to hairpin formation (Gusarov and Nudler 1999). Several models have been proposed about the detailed mechanism by which the terminator RNA hairpin, along with the T residue stretch, leads to transcription termination at Rho-independent terminators (Santangelo and Roberts 2004; Larson et al. 2008; Peters et al. 2011). These in vitro studies, which were performed using various terminators including those possessing a discontinuous T stretch disrupted with other nucleotides, suggest that different mechanisms operate depending on terminators. The sRNA genes and their variants, possessing a long continuous T residue stretch with different stability/structure of the hairpin, would be useful to understand more deeply the roles of the terminator hairpin and the T residues stretch in transcription termination.

Although a number of analyses have been performed regarding the mechanism of transcription termination, little has been known about how the hairpin stability affects the termination position at Rho-independent terminators. We have addressed this question experimentally in this study and demonstrated clearly that the strength of the terminator hairpin controls the termination position within the polythymidine stretch. In other words, the terminator hairpin determines the length of the poly(U) tail of the transcripts depending on its thermodynamic stability. Thus, the control of the termination position by the terminator RNA hairpin is a critical event for the biogenesis of functional sRNAs. It remains to be studied whether the function of many other transcripts including mRNAs is affected by the length of the poly(U) tail. The stronger RNA hairpin would be more beneficial for efficient transcription termination and, therefore, for efficient generation of termination products such as mRNAs in many genes. It is apparent that this is not the case for transcription termination at the Rho-independent terminators of sRNA genes. The thermodynamic stability of the terminator hairpin of sRNA genes is not too strong to ensure the generation of transcripts possessing a long poly(U) tail by preventing premature termination and also not too weak to prevent too much readthrough. Thus, the terminator of sRNA genes is optimized to generate efficiently functional sRNAs. How the stability of the terminator hairpin controls the termination position is certainly an important question to be addressed for understanding the molecular mechanism of transcription termination at Rho-independent terminators. In this connection, it is interesting to note that shortened transcripts were generated at a Rho-independent terminator in an in vitro transcription system with a low concentration of NTPs (McDowell et al. 1994).

Another interesting question regarding the mechanism of transcription termination is how the stress enhances the termination. We demonstrated previously that stress conditions lead to the enhancement of termination at not only sRNA genes but also a gene encoding an mRNA (Morita et al. 2015). It is interesting that low NTP concentrations are reported to enhance transcription termination at Rho-independent terminators in vitro (Reynolds et al. 1992). In addition, it is reported that shortened transcripts were generated at a Rho-independent terminator in vitro when a low concentration of NTPs was used (McDowell et al. 1994). Therefore, one plausible effect of stress is to reduce the level of NTPs in cells.

The simplest type of Hfq binding module consists of a long poly(U) tail, terminator hairpin, and an internal U-rich sequence just before the hairpin (Ishikawa et al. 2012). The poly(U) tail binds to the proximal face of the Hfq hexamer, whereas the internal U-rich sequence preferentially binds to the rim of the Hfq hexamer (Sauer and Weichenrieder 2011; Sauer et al. 2012; Schu et al. 2015). Thus, the roles of the poly(U) tail and internal U-rich sequence in Hfq binding are clear. On the other hand, it remains to be studied whether and how the terminator RNA hairpin is involved in Hfq binding. We showed previously that SgrS variants retain the ability to bind to Hfq and to repress the target *ptsG* mRNA even when the sequence corresponding to the stem–loop structure was dramatically changed as far as the hairpin structure was maintained (Otaka et al. 2011). It is interesting to note that a hairpin-less RNA possessing a base-pairing region and a poly(U) tail is able to base pair with a beacon probe RNA in the presence of Hfq in vitro (Panja et al. 2013). In addition, a structural analysis demonstrated that the hairpin structure seems to be not involved in interactions with Hfq (Dimastrogiovanni et al. 2014). These results suggest that the terminator RNA hairpin plays no role in Hfq binding itself. Further studies are needed to know whether the hairpin structure of the terminator hairpin plays any roles in Hfq binding.

## MATERIALS AND METHODS

### Bacterial strains and plasmids

The *E. coli* K12 strains and plasmids used in this study are listed in Table 1. IT1568 (W3110*mlc*^−^) was used as a parent strain. To construct TM894, TM895, and TM896, the alleles Δ*pnp*::*cat*, Δ*rnb*::*cat* and Δ*rnr*::*cat* of ECK3152, ECK1281 and ECK4175, respectively, were moved to TM542 (Kawamoto et al. 2005) by P1 transduction. ECK3152, ECK1281, and ECK4175 were donated from H Mori, NAIST (pers. comm.). The *rpoC-Flag-cat* allele was constructed according to the modified Datsenko–Wanner protocol using pSU313 harboring the *Flag-cat* sequence (Uzzau et al. 2001). To construct TM905 in which the chromosomal *rpoC* gene was replaced with the *rpoC-Flag* encoding carboxy terminally Flag-tagged β′, the *rpoC-Flag-cat* allele was moved to IT1568 by P1 transduction. TM908 was constructed by removing the *cat* gene flanked by two FRT sequences from TM905.

The DNA primers used are listed in Table 3. Plasmid pSgrS-S-LS1-*rplL*T was constructed as follows: pSgrS-S-*rplL*T was used to amplify the DNA fragment containing the *sgrS-S-LS1* sequence and *rplL*T sequence with primers 1585 and 1544. The amplified DNA fragment was digested with XbaI and HindIII, and cloned into pAraX. Plasmids pSgrS-S, pSgrS-S-LS1, pSgrS-S-LS2, pSgrS-S-LS3, and pSgrS-LS4 were constructed as follows: pSgrS (Otaka et al. 2011) was used to amplify the DNA fragment containing the *sgrS-S*, *sgrS-S-LS1, sgrS-S-LS2, sgrS-S-LS3*, or *sgrS-S-LS4* sequence with primers 1127 and 1839, 1840, 1923 or 1924, respectively. The amplified DNA fragments were digested with XbaI and HindIII, and cloned into pAraX. Plasmids pSgrS-S (*tac*) and pSgrS-S-LS2 (*tac*) carrying the *P*_*tac*_-*sgrS-S* and *P*_*tac*_-*sgrS-S-LS2,* respectively, were constructed as follows: pSgrS-S or pSgrS-S-LS2 was used to amplify the DNA fragment containing the *P_tac_-sgrS-S* or *P*_*tac*_-*sgrS-S-LS2* sequence with primers 1919 and 1839 or 1841, respectively. The amplified DNA fragments were digested with XbaI and HindIII, and cloned into pAraX. Plasmids pRyhB-LS1 and pRyhB-LS2 were constructed as follows: pRyhB (Otaka et al. 2011) was used to amplify the DNA fragment containing the *ryhB-LS1* or *ryhB-LS2* sequence with primers 1144 and 1845 or 1844, respectively. The amplified DNA fragments were digested with XbaI and HindIII, and cloned into pAraX.

**TABLE 3.**
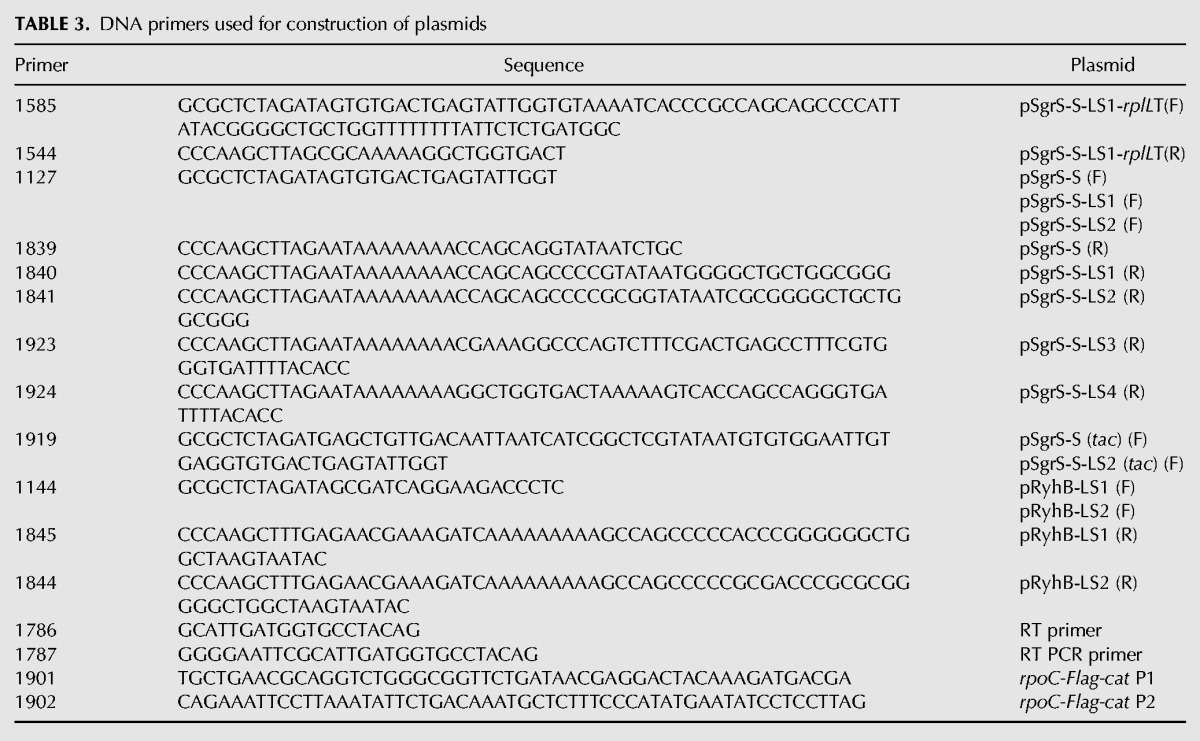
DNA primers used for construction of plasmids

### Northern blotting

Cells carrying the indicated plasmids were grown at 37°C to mid-log phase in LB medium supplemented with kanamycin (15 µg/mL) and indicated amounts of arabinose and αMG when needed. Total RNAs were isolated as previously described (Aiba et al. 1981). To detect SgrS-S and RyhB RNAs, RNA samples were resolved by 12% and 10% polyacrylamide gel electrophoresis, respectively, in the presence of 8 M urea and blotted onto a Hybond-N^+^ membrane (GE Healthcare). To detect tmRNA, *ptsG* mRNA, and *sodB* mRNA, RNA samples were resolved by 1.5% (tmRNA) and 1.2% (mRNAs) agarose gel electrophoresis in the presence of formaldehyde and blotted onto a Hybond-N^+^ membrane (GE Healthcare). The RNAs were visualized using digoxigenin (DIG) reagents and kits for nonradioactive nucleic acid labeling and a detection system (Roche Applied Science) according to the procedure specified by the manufacturer. The RNA probes SgrS-S and RyhB corresponding to the antisense of portion (+168 to +198) of *sgrS* and portion (+1 to +55) of *ryhB*, respectively, were prepared by the DIG RNA Labeling Kit (Roche Applied Science). The following DIG-labeled DNA probes were prepared by PCR using DIG-dUTP: a 363-bp fragment corresponding to the tmRNA (tmRNA probe); a 305-bp fragment corresponding to the 5′ region of *ptsG* (*ptsG* probe); a 210-bp fragment corresponding to the 5′ region of *sodB* (*sodB* probe). Dyna Marker, RNA Low II (BioDynamics Laboratory Inc.) was used as RNA size markers. The positions of RNA markers were shown on the left of the figures of Northern analysis.

### 3′-RACE

Total cellular RNAs prepared from cells harboring pSgrS-S or pSgrS-S-LS2 were resolved by electrophoresis on a 12% polyacrylamide gel in the presence of 8 M urea. After electrophoresis, the gel pieces corresponding to the full-length and shorter transcripts were cut out from the gel. RNAs were eluted from the gel pieces in buffer containing 20 mM Tris-HCl (pH7.5), 0.5 M ammonium acetate, 10 mM magnesium acetate, 1 mM EDTA, and 0.1% SDS overnight at 37°C. Eluted samples were treated with phenol, precipitated, and washed with ethanol. The precipitants were dissolved in 10 µL of H_2_O (UltraPure DNase/RNase-Free Distilled Water, Invitrogen) containing RNase inhibitor (TOYOBO). RNA samples and 5 mM of RNA linker (/5rApp/ CTGTAGGCACCATCAAT/3ddC/, Integrated DNA Technologies) (Churchman and Weissman 2011) were heated for 2 min at 93°C, and chilled on ice for 1 min. The RNA linker was ligated onto the 3′ end of the RNA samples by RNA ligase 2, truncated (New England BioLabs Inc.) for 1 h at 25°C . The mixture was incubated for 20 min at 65°C, and then treated with phenol, precipitated, and washed with ethanol. The precipitants were dissolved in 10 µL of H_2_O containing RNase inhibitor. cDNA was synthesized by the 3′ Full RACE Core Set (TAKARA) according to manufacturer's instructions with RT primer 1786. The cDNA was amplified with primers 1127 and 1787 by using PCR SuperMix (Life Technologies). Cycling conditions were followed as: 98°C/30 sec; 20 cycles of 95°C/10 sec, 53°C/30 sec, 72°C/60 sec; 72°C/5 min. The amplified DNA fragments were purified by 6% native PAGE, followed by digestion with XbaI and EcoRI, and cloning into pTWV228 (TAKARA). Positive colonies were randomly picked up and plasmid DNAs were extracted using the QIAprep Spin Miniprep Kit (QIAGEN). Inserted cDNA sequences were analyzed with −47 sequence primer using CEQ8000 sequencer (SCIEX).

### Pull-down assay

Cells were grown in 200 mL of LB medium at 37°C. At *A*_600_ = 0.6, 0.1% αMG was added and incubation was continued for 10 min, and then 0.02% arabinose was added and incubation was continued for 5 min. Cells were harvested, and washed by 15 mL STE buffer (100 mM NaCl, 10 mM Tris-HCl at pH 8.0, and 1 mM EDTA). The cells pellet was suspended in ice-cold 1 mL IP buffer 1 (20 mM Tris-HCl at pH 8.0, 0.1 M KCl, 5 mM MgCl_2_, 10% glycerol and 0.1% Tween20). The cell suspension was crushed by µT-01 Beads Crusher (TITEC) with ϕ0.350∼0.500 mm of glass beads, followed by centrifugation at 10,000*g* for 10 min at 4°C. The supernatant (crude extract [CE]) was incubated with 50 µL of anti-Flag M2-agarose suspension (Sigma-Aldrich) in 10 mL of IP buffer 1 for 20 min at 4°C. The mixture was filtered by using a mini chromatography column (Bio-Rad). The agarose beads were washed twice by 10 mL of IP buffer 1. The proteins bound to the beads were eluted with 50 µL of IP buffer 1 containing 0.4 mg/mL Flag peptide (Sigma-Aldrich) and used as bound fraction (B). To analyze proteins, CE (2.5 µL) and B (2.5 µL) were mixed with SDS-PAGE loading buffer (6.25 mM Tris-HCl at pH 6.8, 2% SDS, 10% glycerol, 5% β-mercaptoethanol, 0.1% bromophenol blue). The samples were heated for 5 min at 100°C and subjected to Western blotting using an anti-Flag monoclonal antibody (Sigma-Aldrich). Signals were visualized by the Lumi-light Western Blotting Substrate (Roche). To analyze RNAs, CE (10 µL) and B (10 µL) were treated with phenol, precipitated, and washed with ethanol. Each precipitant was dissolved in 6 µL of RNA buffer (0.02 M sodium acetate, pH 5.5, 0.5% SDS, and 1 mM EDTA). The RNA samples were subjected to Northern blotting using SgrS-S RNA probe.

### In vitro transcription assay

Flag-tagged RNA polymerase (RNAP-Flag) in which the carboxy-terminus of the β′ subunit is tagged with the Flag sequence was purified as follows. TM908 carrying the *rpoC-Flag* allele was grown in 400 mL of LB medium at 37°C. At *A*_600_ = 0.8, cells were harvested and washed with 40 mL of STE, and suspended in ice-cold 4 mL of IP buffer 2 (20 mM Tris-HCl at pH 8.0, 0.2 M KCl, 5 mM MgCl_2_, 10% glycerol, and 0.1% Tween20). The cell suspension was crushed by the µT-01 Beads Crusher (TITEC) with ϕ0.350∼ 0.500 mm of glass beads, followed by centrifugation at 10,000*g* for 10 min at 4°C. The supernatant was incubated with 200 µL of anti-Flag M2-agarose suspension (Sigma-Aldrich) in 10 mL of IP buffer 2 for 30 min at 4°C. The proteins bound to the beads were eluted with 200 µL of IP buffer 2 containing 0.4 mg/mL Flag peptide (Sigma-Aldrich) and concentrated by Amicon Ultra (0.5 mL 30K) Centrifugal Filters (Millipore). The purity and the concentration of RNAP-Flag were estimated by electrophoresis using Blot 4–12% Bis-Tris Plus gel and SimplyBlue SafeStain (Invitrogen) (Fig. 6C). An equal amount of glycerol was added to the concentrated RNAP-Flag solution and used for an in vitro transcription assay. Plasmids pSgrS-S (*tac*) and pSgrS-S-LS2 (*tac*) were purified from cells harboring the respective plasmid by using QIAprep Spin Miniprep Kit (QIAGEN); then they were used as DNA templates for in vitro transcription assay.

Plasmid DNA (1.6 µg) was incubated in 10 µL of 0.1× TE containing 40 mM NaCl for 2 min at 95°C, and then cooled down to 30°C. The DNA was mixed with 4 nM RNAP-Flag and 25 µM NTPs in 20 µL (final volume) of transcription buffer (50 mM Tris-HCl at pH 8.0, 50 mM NaCl, 3 mM MgCl_2_, 0.1 mM EDTA, 0.1 mM DTT, 0.01% BSA) containing RNase inhibitor (TOYOBO). The reaction mixture was incubated for 30 min at 37°C. The reaction was terminated by adding phenol. Nucleic acids in the aqueous phase were precipitated with ethanol. The pellets were washed with ethanol, and dissolved in RNA buffer. The samples were subjected to Northern blotting using the SgrS-S RNA probe.
